# Integrating health promotion with and for older people - eHealth (IHOPe) – evaluating remote integrated person-centred care: Protocol of a randomised controlled trial with effectiveness, health economic, and process evaluation

**DOI:** 10.1186/s12877-023-03866-6

**Published:** 2023-03-27

**Authors:** Zahra Ebrahimi, Emmelie Barenfeld, Hanna Gyllensten, Patricia Olaya-Contreras, Andreas Fors, Eva Fredholm, Joanne M. Fuller, Mahboubeh Godarzi, Birgitta Krantz, Karl Swedberg, Inger Ekman

**Affiliations:** 1 Institute of Health and Care Sciences, Sahlgrenska Academy, University of Gothenburg, Gothenburg, Sweden; 2 Centre for Person-Centred Care (GPCC), Sahlgrenska Academy, University of Gothenburg, Gothenburg, Sweden; 3 Department of Occupational Therapy and Physiotherapy, Sahlgrenska University Hospital, Gothenburg, Sweden; 4 Region Västra Götaland, Research, Education, Development and Innovation, Primary Health Care, Gothenburg, Sweden; 5 Department of Molecular and Clinical Medicine, University of Gothenburg, Gothenburg, Sweden

**Keywords:** Person-centred care, Person-centered care, Patient-centred care, Patient-centered care, Integrated care, Frail older people, Health promotion, Prevention, eHealth, Telemedicine

## Abstract

**Background:**

Healthcare and welfare systems worldwide are unprepared to accommodate the growing population of older people. Simultaneously, the cost of reactive care for older people is increasing. However, healthcare systems in many countries are reforming towards integrated and person-centred care with a focus on health promotion and proactive actions. *The Integrating Health Promotion with and for Older People – eHealth (IHOPe)* project aims to describe and evaluate a person-centred e-support intervention that promotes a sustainable partnership between community-dwelling frail older people and health and social care professionals.

**Methods:**

The IHOPe project is designed as a randomised controlled trial comparing a control group receiving standard care with an intervention group receiving standard care and add-on person-centred care through telephone support and a digital platform. The primary outcome measure is a composite score of changes in general self-efficacy and the need for unscheduled hospital care. The project is conducted in Gothenburg, Sweden. At least 220 participants aged ≥ 75 years will be included after being screened using a frailty instrument. The study design, intervention components, digital platform, and questionnaires were developed in close collaboration with an advisory group of inter-professional researchers, stakeholders, clinicians, and older representatives. Data will mainly be collected through questionnaires at baseline and 3, 6, and 12 months after inclusion in the study. Recruitment is ongoing and should be completed during 2023. Data will be analysed using quantitative and qualitative methods. The evaluation will include effectiveness, process, and health economics. The study was approved by the Regional Ethical Review Board in Gothenburg, Sweden (Dnr 2019–05364, Dnr 2020–03550, Dnr 2021–03255).

**Discussion:**

The findings will expand our knowledge of remotely integrated person-centred care for frail older people. Thereby, the IHOPe project is expected to fill highlighted knowledge gaps on intervention evaluations including the triad of person-centred, digital, and integrated care elements, as well as economic evaluations of remote health services for frail older people. The study is ongoing, and the results are not completed but if they turn out to be positive, implementation is not limited to time or location.

**Trial registration:**

ClinicalTrial.gov: NCT04416815. Registered 07/06/2021.

## Background

By 2050, the number of people aged ≥ 75 years is expected to increase by 50% worldwide [1, 2], which means an escalating global demand for diverse healthcare systems [3, 4]. Moreover, the number of people aged > 80 years is also increasing rapidly [1, 2]. Advanced age is often associated with increased risk of frailty, multi-morbidity, and functional impairments [5]. Thus, the development of innovative approaches with health promotive and proactive actions is an urgent need to achieve a sustainable healthcare system that is efficient, equal, and supports health in frail older people [3, 6, 7]. Such approaches should incorporate older people’s capabilities and strengthen a preventive approach in healthcare services, as well as reduce complexity and improve accessibility to health planning and care coordination from the patient perspective [6, 8]. Implementing person-centred care (PCC), utilization of accessible digital health services [2] and teamwork [9] are keystones in such a healthcare system redesign [7, 10]. Several studies show that PCC can be delivered remotely [11] but needs further development for enabling remote teamwork with and for frail older people [12]. Therefore, the project *Integrating Health Promotion With and for Older People - eHealth (IHOPe)* focuses on implementing health planning based on a person-centred ethic [13, 14] and capability approach [15] together with frail older people by working as a team through telephone support and a digital platform.

**Frailty** is a complex syndrome, distinguished from but interrelated to disability and co-morbidity [5, 16, 17]. Frailty is characterised by loss of function and physiological reserve capacity, increased risk of acute illness, falls, disability, institutionalisation, and death [5, 17]. Moreover, frailty in older people is linked to the degree of functional disability regarding their capacity to accomplish daily activities [18, 19]. Frail older people often have more diffuse symptoms than younger people, making diagnosing and identifying underlying symptom causes more difficult. The complexity of detecting frailty in older people can lead to unnecessary emergency visits, as the underlying cause of the health disruption has not been addressed. Identifying frail older people where the need for emergency care could be prevented by frailty screening is necessary to decrease the high number of emergency care visits in this patient group [16, 20].

PCC emphasises the relationship between the healthcare professional (HCP) and the older person as a prerequisite for shared decision making in health planning and successful care [21]. In line with our previous research, person-centred telephone support can be seen as a promising tool to initiate health planning with older people [11]. In addition, digitally shared documentation indicated an increase in self-efficacy [22, 23]. Self-efficacy, defined as a person’s belief that they can successfully execute behaviours necessary to achieve desired health goals [24], has been proposed as a central concept in PCC [25]. PCC aims to co-create patients’ self-efficacy rather than convince or educate them about the value of such behaviours [14, 24]. Three key components in implementing PCC in daily clinical practice are formulated to build self-efficacy [14]. The first step is to initiate the partnership by capturing patients’ narratives and experiences of their opportunities and barriers in everyday life. The next step is to work the partnership between patients and HCPs through discussion and shared planning of care and treatment. The last step is safeguarding the partnership by documenting the patient’s preferences, beliefs, values, and agreement on future planning [14]. These three steps of initiating, integrating, and safeguarding the partnership are incorporated into this project to operationalise PCC.

Person-centred teamwork involves the older person as an equal partner in the health care team, in which HCPs work *with* the older person (and often with significant others) [9]. Previous research on supporting frail older people has had their point of departure in existing healthcare teams (usually situated within social and healthcare organisations) [26–29]. In addition, the patients and their significant others have not been described as team members. The need to engage frail older people as equal partners in the healthcare team has been recently highlighted [9]. The partnership-building process may be facilitated through person-centred telephone support and an accessible digital platform. Our research has shown that working in partnership remotely is possible and efficient [11, 30]. To our knowledge, no previous research has investigated the effectiveness of such support among frail older people. The innovative aspect of this study is the remote use of a preventive strategy by co-created health planning between frail older people and their health and social care team. The underlying hypothesis is that working in partnership through telephone support and the opportunity to communicate with HCPs via the digital platform is a feasible and effective use of available resources. Such an intervention will lead to reduced hospital admissions and postpone a decrease in self-efficacy in frail older persons. In addition, the project will expand our knowledge about if and how a remotely person-centered intervention can contribute to bridging the digital exclusion experienced by frail older people in many of today’s eHealth solutions [31, 32].

## Aim

The **IHOPe** project aims to evaluate the effects, describe the process, and perform a health-economic evaluation of a person-centred remote intervention to promote a sustainable partnership between community-dwelling frail older people and health and social care professionals.

**Specific aims**:

- To evaluate the preventive effects of a remote person-centred support on self-efficacy and hospitalisation of community-dwelling frail people ≥ 75 years.
- To describe, through a process-evaluation, the applicability, feasibility and reach of remote PCC.
- To explore and evaluate frail older peoples’ experiences of a remote person-centred intervention.
- To perform a health economic evaluation of the remote PCC intervention compared to usual care.

## Methods and design

The IHOPe study will be designed as a randomised controlled trial (RCT) with two parallel groups and a primary endpoint assessed three months after inclusion. In addition, the IHOPe project will include health economic and process evaluations. The project will be a complex intervention and, as such, features a multitude of influencing factors [33]. The study design will be guided by the revised Medical Research Council’s (MRC) framework for complex interventions [34] and the guidelines for reporting parallel group RCTs [35]. We opted for the updated version of the original MRC framework due to its iterative approach and increased focus on early-phase piloting and development of the complex intervention [34]. The IHOPe project will consist of two phases corresponding with the three steps of *Development and Feasibility and Piloting* and *Evaluation* of the MRC framework. The *implementation* steps will be planned throughout the project. This manuscript presents the RCT.

Phase 1: Develop and test the feasibility of a person-centred e-support intervention and pilot the RCT design.

Phase 2: Evaluate the effects, describe the process, and perform a health economic evaluation of the person-centred e-support intervention.

### Patient and public involvement

The involvement of different categories of knowledge users (e.g. older people, health professionals and decisionmakers) is advocated as a crucial part of research [36, 37]. Therefore, the planning and designing of the study have been conducted in close communication and collaboration with an advisory group including older people, inter-professional researchers, decisionmakers, and clinicians. The development of the digital platform was guided by a participatory design [38]. Moreover, the entire study, including recruitments, choice of instruments, questionnaire development, and the intervention components, have been, and continually will be, co-created with the advisory group.

Participants and setting.

The IHOPe project will be conducted in Sweden and targets community-dwelling older people aged ≥ 75 years, their families, and professionals working in social and healthcare services. Sweden has an ongoing redesign of healthcare services towards person-centred integrated care, which aims to strengthen primary care services and self-care [10]. Health costs in medical care are mainly financed through taxes, where municipalities are responsible for services for older people according to assessed needs. Several actors could be involved in formal care activities for frail older people representing different health and social care organisations. There is also an option to seek private health and social care providers [10]. In addition, it is common that older people are receiving support or help from informal caregivers [3, 10]. Therefore, the evaluation process also includes significant others and HCPs invited to the IHOPe platform. All participants must provide signed informed consent before any study procedures occur.

#### Inclusion criteria

Inclusion criteria will be national registration within the Region Västra Götaland, men and women aged ≥ 75 years living in ordinary housing and screened as frail at an emergency department but not hospitalised.

#### Exclusion criteria

Exclusion criteria will be those needing palliative care in the final stages of life, no registered address, participating in a conflicting randomised study, or cognitive dysfunctionality (not oriented to time, place, and person).

### Enrolment and randomisation

**Fig. 1:**
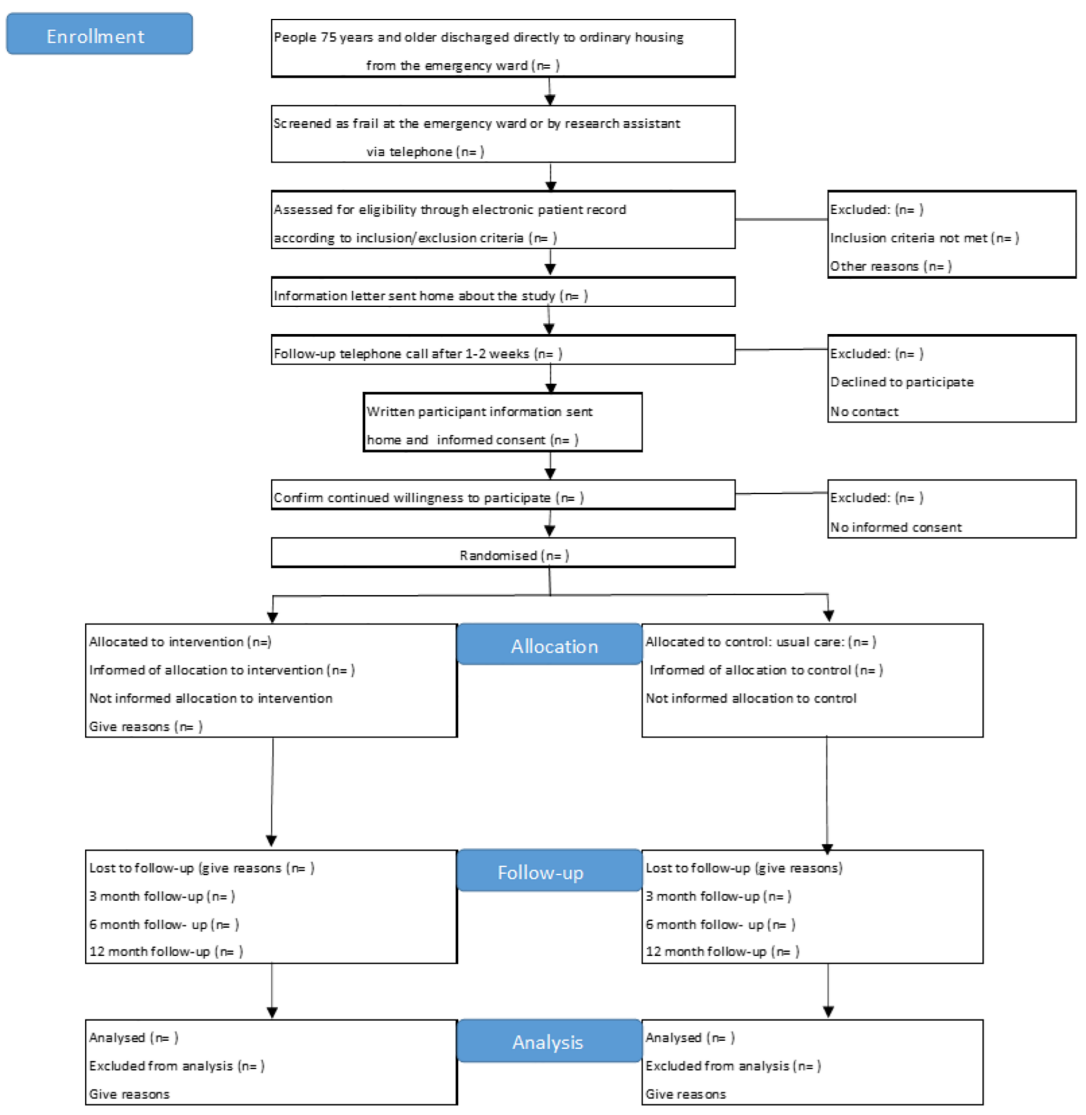
Flow chart of participant enrolment, allocation, follow-up, and analysis [35]

Frail older people will be recruited from the emergency department at a university hospital in Gothenburg, Sweden, to include a diverse population of frail older people. The planned flow of participants is shown in Fig. 1.

Participants aged ≥ 75 years will be screened using an instrument developed by the Frail Elderly Support Research Group (FRESH) [39] as part of usual care at the emergency department or by a research assistant via telephone. The screening results (if frail or not) will be documented. The research assistant will screen patients identified as frail for eligibility to participate in the study using the above-mentioned inclusion and exclusion criteria and the electronic patient record. A study information letter and invitation to participate will be sent to eligible patients within two weeks after their emergency visit. The patient will then be contacted by telephone to provide verbal information and asked about consent to participate. Finally, written participant information and informed consent forms will be sent to patients willing to participate.

All who fulfil the inclusion criteria and consent to participate will be randomised into the study. Participants will be randomly assigned to the control or the intervention group with a 1:1 allocation using a computer-generated randomisation schedule. The control group will receive usual care whilst the intervention group will receive the person-centred e-support intervention for six months in addition to usual care.

The recruitment of participants to the main study started after the inclusion of 17 participants in a pilot study. The full-scale RCT is currently ongoing. Follow-up for each study participant continues for 12 months from inclusion.

Recruitment of significant others and HCPs depart from the older people’s network by contact information to significant others or formal carers invited to the intervention.

### Control group

The participants allocated to the control group will receive *usual care*. Routine care for older people in Sweden includes care delivered by diverse HCPs in hospital, primary care, municipal, and home care services. Also, informal carers are often involved. It is unusual to have any follow-up contact from the emergency ward for frail older people who are not admitted to hospital. However, within usual care some patients are contacted by a ‘mobile team’ (consisting of nurses and physicians) through a coordinator at the emergency ward. The mobile team visits the patient at home or contacts the municipal care provider for further follow-up, depending on the older person’s healthcare needs and status [40]. However, the mobile team does not deliver digital healthcare.

### Intervention group

In addition to usual care, participants allocated to the intervention group will receive person-centered telephone support and the opportunity to communicate with HCPs through the digital platform for six months. The intervention is delivered remotely and is coordinated by dedicated registered nurses (RNs). The HCPs conducting the intervention receive continuous training through workshops with specialists in PCC, ethics, communication, and pedagogics on how to apply remote PCC.

Intervention components.

The intervention aims to initiate partnership-building, encourage older people to describe their health situation and needs, and further identify and engage their capabilities and resources [43].

### Telephone support and the creation of a health plan

An HCP will initiate the first telephone conversation shortly after study inclusion (within two weeks). After the first telephone call, a follow-up appointment will be mutually planned and agreed upon between the HCP and the older person. In addition, there is the opportunity for incidental contact with the older person by telephone or through the digital platform. The participants’ narrative of their everyday situation, as well as their needs, resources, and health-related goals are the core components of the telephone conversations [11, 41]. The conversation will start with recapitulating the aim of PCC support, asking open-ended questions, and listening to the participants’ experience of their condition, context, needs, and resources. The HCP will confirm and summarise the content of the conversation and, in collaboration with the participant, co-create achievable and relevant health-related goals. The participants’ narratives (at times in partnership with significant others), goals, and strategies to manage everyday life, as well as needs of support and actions and resources to achieve agreed health-related goals, will then be documented in a health plan by either the participant or the HCP and uploaded to the digital platform. This health plan is, however, a living document and can be modified and reformulated during the intervention, as is described in other PCC projects with different populations [22, 42, 43].

### The digital platform

The digital platform has been tested in previous studies and the present study will apply the same procedure of working together via the platform [22, 43]. All participants are invited to communicate on the platform. The participants or the responsible HCP will upload the mutually agreed health plan to the digital platform. Depending on agreements during the phone call, family, friends, and additional health professionals may be invited to join the digital platform team. An HCP will introduce and explain how to use the digital platform and create a personal account. The platform is a mediator through which the participants, together with their team (i.e., formal and informal family and informal carers), can develop the health plan and follow-up actions and goals. The platform allows self-monitoring through daily ratings on a scale from 1 to 5 on symptoms and general well-being (e.g., how well they have slept). In addition, the participants and the HCPs can communicate through messages in a chat-like forum. The platform will contain an assembly of links to other relevant web pages that the participants can use to seek information or connections. HCPs log into the platform on weekdays to be updated on patients’ activities and check for messages [22, 43]. If signs of deterioration occur needing for example changes in the pharmacological treatment, the participants will be advised to contact their primary care physician and if symptoms then are assessed as urgent, they are encouraged to contact the emergency healthcare services.

### Data collection

Various data sources will be used, including questionnaires (see detailed description in Table 1), activities performed by the participants in the digital platform, time consumption during conversations, and for writing health plans and medical records. In addition, the selection of participants (in the intervention group), as well as significant others and health professionals invited for teamwork, will be asked if they are willing to participate in focus groups or individual interviews. Clinical and self-reported data (e.g., questionnaires) will be collected at baseline and after 3, 6, and 12 months. The primary and secondary endpoints have been used in large national or international studies and tested for validity and reliability [42, 44]. Moreover, register data will be collected retrospectively from the National Board of Health and Welfare: each registered holder, health care encounters from the regional patient register VEGA [45], use of prescribed drugs dispensed in outpatient pharmacies from the Swedish Prescribed Drug Register (SPDR) [46], use of home help services and supported living facilities, municipal health care use, and information about potential causes of death.

### Outcomes and measurements

Questionnaire data will be gathered at baseline and after 3, 6, and 12 months from the inclusion date. Self-reported baseline characteristics of the participant’s sex, age, civil status, country of birth, and level of education will be collected through questionnaires at baseline.

### Primary outcome

In line with previous RCTs evaluating PCC [22, 43] the primary outcome will be a composite of clinical changes [47], in general self-efficacy and the need for hospital care for unscheduled reasons after 3 months. Each participant will be classified as improved, deteriorated, or unchanged at three months as follows:

- *Deteriorated*: the participant’s general self-efficacy has decreased by $\ge$5 units or has been admitted to hospital for unscheduled reasons two or more times.
- *Improved*: general self-efficacy has increased by $\ge$5 units, and the participant has not been admitted to hospital more than once.
- *Unchanged*: the patient has neither deteriorated nor improved.

### Secondary outcomes

A composite of changes in general self-efficacy [48, 49] and the need for hospital care for unscheduled reasons at 6 and 12 months.

#### General self-efficacy

The General Self-Efficacy Scale (GSES) [48, 49] is a 10-item self-assessment psychometric questionnaire designed to measure a broad and stable sense of personal competence to deal with stressful situations. Ratings are made on a 4-point Likert scale (1 = not at all true, 2 = hardly true, 3 = moderately true, 4 = exactly true) and are summed to give a total score ranging from 10 to 40, with higher scores indicating generalised greater self-efficacy. The GSES has been validated in several languages. An increase of 4.6 units in the total score has been proposed and is used as a limit for a minimally important difference [11, 22, 41].

#### Hospitalisation

Hospitalisation refers to the number of unplanned emergency department visits and hospital admission according to patients’ self-reported responses (questionnaires) and data in medical records.

#### Health-related quality of life by EuroQol 5 dimensions health state questionnaire (EQ-5D) and quality adjusted-life years (QALY)

EQ-5D [50, 51] is a generic measure of health status consisting of five dimensions (mobility, self-care, usual activities, pain/discomfort, anxiety/depression), each with responses indicating three levels of severity (no problems, some or moderate problems, extreme problems). The EQ visual Analogue scale (EQ-VAS) is a standard vertical 20 cm VAS recording people’s rating of their current health-related quality of life (HRQoL) ranging from ‘the best health you can imagine’ to ‘the worst health you can imagine’ [50, 51]. The EQ-5D index will be used to derive QALY using the Swedish experience-based value set [52] and an area-under-the-curve calculation [53]. An alternative valuation using a general population value set from the UK [54] will be part of the sensitivity analysis, as Sweden has no such societal valuation.

#### ICEpop CAPability measure for older people (ICECAP-O) and capability-adjusted life years (CALY)

The ICECAP-O capability index [55, 56] estimates quality of life (QoL) in a broader sense compared to the HRQoL, including five attributes (attachment, role, enjoyment, security, control), each with four response levels. The ICECAP-O instrument will be used to derive CALY using the British valuation [56].

#### Change in daily activities based on the Activities of Daily Life (ADL) staircase

Change in dependence in daily activities will be assessed based on the ADL staircase [57, 58]. The Instrument contains a cumulative scale of six personal activities (P-ADLs: bathing, dressing, going to the toilet, transfer, continence, feeding) and four instrumental activities (I-ADLs: cleaning, shopping, transportation, cooking). Dependence is defined as receiving personal or directive assistance from another person [57, 58]. In line with previous studies [59, 60] evaluating ADL in frail older people, a participant living with another person is assessed as “independent” if they are capable of performing the activity without assistance when alone.

#### Societal costs

Resource use in the economic evaluation will include register data on health care use (held by the Region Västra Götaland), as well as dispensed drugs, and use of social care use (held by the National Board of Health and Welfare). Informal care and other expenses to the participant and family and friends related to the treatment will be collected using patient questionnaires and based on user data from the digital platform. Costs (both reimbursements and out-of-pocket costs) for prescribed drugs will be obtained from the Swedish Prescribed Drug Register (SPDR) [46]. The out-of-pocket costs for healthcare use and social care will be calculated from national statistics on patient fees while accounting for the national high-cost protection schemes, in line with previous work [61]. Regional costs for healthcare will be calculated using cost-per-patient data and diagnosis-related group weights (DRG) from specialised care and the national mean cost for producing one DRG, while the corresponding costs for primary care and social care will be derived using national cost statistics. Additional questions have been added in the questionnaire to identify expenses not accounted for in register data. Informal care costs will be viewed as a direct cost, and thus valued at the average wage and social security contribution of employing a formal caregiver, sometimes called the replacement cost approach [62].

#### Incremental cost-effectiveness ratio (ICER)

The ICER will be calculated as the societal cost difference between groups divided by the corresponding difference in QALY [52] and CALY [56], respectively as recommended in studies including both health and social services [63, 64].

#### Change in the burden of medicine use in everyday life based on The Living with Medicines Questionnaire version 3 (LMQ-3)

Participants will self-rate eight domains: relationship with health professionals, practical difficulties, interference with daily life, lack of effectiveness, side effects, general concerns, cost, and lack of autonomy. Each domain will be rated on a 5-point Likert scale (strongly agree to strongly disagree) [65].

#### Change in the participants’ self-rated overall level of burden of medicine

Participants will assess the overall level of burden of medicine on a 10 cm VAS from 0 (no burden at all) to 10 (extremely burdensome) [65].

#### Social network, social support, and loneliness

Five items will ask about social network, sufficient support, someone to trust and confide in, experience safety and security and feelings of loneliness [66–68].

#### Self-reported frailty indicators according to the Tilburg Frailty Indicator (TFI)

The TFI [67, 68] will be used to assess self-reported frailty indicators, unexplained weight loss, difficulty in walking, strength in hands, physical health, physical tiredness, balance, problem with hearing and vision (physical domain), cognition, depressive symptoms, anxiety, and coping (psychological domain), living alone, social relations and social support (social domain). An additional question about falls during the past 3 months will also be used [66].

#### Frailty according to the clinical Frailty Scale (CFS) version 1.2

The degree of frailty will be assessed from 1 to 9; 1: very fit, 2: well, 3: manging well, 4: vulnerable, 5: mildly frail, 6: moderately frail, 7: severely frail, 8: very severely frail, 9: terminally ill) [69].

For details about primary and secondary outcomes, data sources, and time points for data collection, see Table 1.

**Table 1:** Overview of primary and secondary outcomes, demographic measures, data sources, and time points for data collection

	Research assessment/data source/register data	T0 baseline	T1 3 months	T2 6 months	T3 12 months	Retrospectively collected
Primary outcomes						
A composite of clinical changes in general self-efficacy and the need for hospital care for unscheduled reasons at 3 months	Questionnaires	x	x			
	Medical records from baseline up to 3 months and register data					
**Secondary outcomes**						
A composite of changes in general self-efficacy and the need for hospital care for unscheduled reasons at 6 and 12 months	Questionnaires	x		x	x	
	Medical records from baseline up to 12 months and register data					
Health-related quality of life	Questionnaires	x	x	x	x	
Capability-related quality of life according to ICECAP-O	Questionnaires	x	x	x	x	
Hospitalisation	Questionnaires, Medical records					x
General self-efficacy	Questionnaires	x	x	x	x	
Change in ability to perform activities of daily living according to the ADL staircase	Questionnaires	x	x	x	x	
Societal costs	Register data, Questionnaires, Digital platform data, and Communication lists.					x
Incremental cost-effectiveness ratio	Questionnaires Registers	x	x	x	x	x
Change in the burden of medicine use in everyday life according LMQ-3	Questionnaires	x	x	x	x	
Change in the participants’ self-rated overall level of burden of medicine	Questionnaires	x	x	x	x	
Social network, social support, and loneliness five questions	Questionnaires	x	x	x	x	
**Demographics**		x				
Background questions, six questions	Questionnaires	x				
Self-reported frailty according to the TFI	Questionnaires	x				
Frailty according to CFS	Researchers’ objective assessment	x				

ICEpop CAPability measure for Older people (ICECAP-O), Activities of Daily Life (ADL), Living with Medicines Questionnaire version 3 (LMQ-3), Tilburg Frailty Indicator (TFI), Clinical Frailty Scale (CFS).

### Blinding

The nature of the intervention means that neither participants nor the HCPs in the IHOPe intervention can be blinded to allocation in the RCT.

### Sample size and power calculation

In the full-scale RCT and health economic evaluation a minimum of 220 patients must be randomised to the two arms based on the primary outcome measure. To achieve 80% power based on a p-value of 0.05 and allow an increase in the proportion of improved or unchanged patients from 20 to 40%, 91 participants in each group (control and intervention) are required. We plan to include 110 patients in each group to have a comfortable margin for dropouts and withdrawals.

### Timeline

Recruitment of participants started in the spring of 2020 and is expected to be completed in 2023. The intervention will continue until early 2024. Data for the primary outcome will be collected three months after the last patient inclusion. Data collection for the secondary outcome measures will continue until early 2025. After that, retrospective register data will be obtained as there is a delay from data collection to transmission to the administrative registers.

### Data analysis

Analysis will be performed according to two analysis sets: (1) the intention to treat set, meaning that all allocated participants will be analysed in the group to which they were randomised [70] and (2) the per-protocol set, which includes only those participants who at least have one telephone conversation and an agreed health plan to indicate an expected minimum level of participation.

If necessary, eventual differences between the intervention and control group at baseline will be adjusted for in the analysis. Different options for handling missing data will be considered based on the collected data. Missing data due to death will be replaced with a value for worst-case change (deteriorated in the composite of primary outcomes). Based on the assumptions that the study sample is expected to decline over time due to the ageing process and that deteriorated health is a common reason not to fulfil follow-ups, the imputation model of median change deterioration will likely be used. However, the final decision on how to handle missing data will be decided by the research team in consultation with a statistician.

Descriptive and analytical statistics will be used to compare the control and intervention groups and to measure change over time. The chi-square or Fisher’s exact test will be computed to compare the proportions between groups. Logistic regression will be used to calculate odds ratios (ORs) with 95% confidence intervals (CIs). Parametric tests will be performed when suitable, and non-parametric tests will be applied when analysing ordinal data.

### Health economic evaluation

Reporting of the economic evaluation will follow the Consolidated Health Economic Evaluation Reporting Standards [71]. Costs will be described and analysed as cost components and total cost, with 95% CIs to indicate uncertainty. Bootstrapping will be used to calculate CIs for any skewed variables [72]. The distribution of costs will also be analysed by major stakeholders: county council/regions, municipalities, market sectors (productivity loss), and individuals/family/friends.

In the case of clearly beneficial effects for costs and intervention outcomes (i.e., lower costs and more health created compared to the alternative) for one of the treatment arms, thus dominating the comparator, this information will be reported. However, increased health benefits are often associated with higher costs, in which case we will use cost-utility analysis to estimate the cost of gaining one additional QALY or CALY, respectively. According to state-of-the-art practice, sensitivity analyses will be applied to test the robustness of the results to necessary assumptions and alternative cost levels. A probabilistic sensitivity analysis will be conducted using longitudinal regression analysis [73] with bootstrapped samples that are visualised using a cost-effectiveness plane. This regression analysis enables adjustment for identified confounding factors. Results from the regression analysis will be compared to the corresponding estimations made using multiple imputations, which have been used more often in previous studies [74].

In addition, group-based trajectory modelling will explore potential cost variations and outcomes within the patient group [75]. This method identifies groups of patients with a similar trajectory in some outcome over time, such as health care costs. The identified groups will then be used to explore patient characteristics associated with such trajectories to provide guidance on which patients would benefit initially from a more intense intervention or are more likely to deteriorate.

### Process evaluation

Several sub-studies will be conducted to widen and deepen the understanding of the impact of the intervention mechanisms. The following questions will guide the evaluation: How do frail older people experience the intervention? To what extent and how meaningful is the intervention to frail older people? To what extent will the intervention reach frail older people? Where applicable, additional analyses describing costs during the implementation phase will be conducted [76].

A mixed-method approach will be applied to deal with the intervention’s complexity by combining quantitative and qualitative methods. Quantitative data will be collected from questionnaires and ratings of the telephone and platform support, which will be attached to the 6-month questionnaire and sent to participants in the intervention group. We will also analyse data on using different platform functions and the number and modes of contact between HCPs, participants, and team members invited to the platform during the intervention period. The content of audio-recorded person-centred conversations and written health plans will also be analysed. In addition, qualitative interviews will be performed to gain a better understanding of the intervention mechanism of impact from the participants’ and their team members’ perspectives. However, in the process evaluation the number of informants depends on the research question in the specific sub-study. There will be a purposeful sampling of participants and informal and formal caregivers for interviews. Qualitative data will be analysed using content analysis and quantitative data will be analysed using descriptive statistics.

### Ethics and dissemination

An ethical application has been approved by Swedish Ethical Review Authority (Dnr 2019–05364, Dnr 2020–03550, Dnr 2021–03255), and the study will comply with the ethical principles of the Declaration of Helsinki. All participants will be asked to sign a written consent form after receiving oral and written information about the study. Informed consent will also be asked of participants accepting to be interviewed.

The findings will be disseminated through academic publications and conferences in the field of health care and medicine. Moreover, the results will be presented for health and social care professionals through appropriate local forums. A user-friendly summary of findings will be sent to participants who have indicated they are interested in the results.

## Discussion

Nearly 40% of patients who seek the emergency ward are frail older people. Identifying those screened as frail but are directly discharged to home is crucial because they risk “falling between the chairs” [3, 20]. Frail older people need more focused health-promoting actions to prevent hospitalisations and promote well-being [20]. The IHOPe project focuses on health promotive actions based on PCC and the capability approach, which adds an essential component to existing evidence-based teamwork approaches, such as comprehensive geriatric assessment or case-manager interventions that focus on providing care. Barriers reported in the implementation of person-centred practice is the lack of crucial ethical underpinnings for PCC, including the patient being an equal team partner [13, 77]. In IHOPe two persons representing frail older people collaborated with the research group in designing the project. The intervention and connecting to the theoretical assumptions have been emphasised: for example, the older person being an equal partner and health and social care professionals working *with* the older person (and their significant others), also acknowledging their social world and internal and external capabilities [14, 21, 78]. This person-centred remote support and conversations based on frail older peoples’ narratives will facilitate reviewing their everyday life from their perspectives and elucidating and confirming their resources and strategies to manage the consequences of frailty, which may lead to retained or improved self-efficacy.

In addition to person-centred telephone communication, the partnership with frail older people will be enhanced through a digital platform, a challenging and essential aspect of the intervention that facilitates the inclusion of individuals living in digital alienation. Previous or ongoing studies, including health planning after frailty screening, have mainly evaluated effects on interventions in existing care teams focusing on how professionals work for older people in different settings face-to-face [27, 29, 79].

A person-centred approach correlates to improved work satisfaction among professionals [80]. Opportunities for teamwork with frail older people will be enhanced, leading to less stress and effective use of the efforts of HCPs and informal carers. IHOPe aims to directly contribute to reaching the national goals of persons ageing at home. Through modest adjustments, IHOPe can be easily applied to existing healthcare systems. The possibility to upscale is in line with the national eHealth strategy, with IHOPe representing a workable asset that is not limited to time or location. Consistent with person-centred ethics, this is the first study of PCC that includes a capability approach in the economic evaluation [81].

Some research indicates that PCC could effectively use resources in care, even remotely delivered [82]. However, even if remote health-promoting services are recommended to improve reach [83], access, and efficiency in health care, they are underused in older populations [84]. Thus far, studies evaluating remote interventions have evaluated interventions performed at a late stage, when people are already highly dependent on help. A combined scoping and systematic review [85] found that few studies evaluated proactive interventions involving the triad of person-centred, digital, and integrated care elements. Therefore, this project will include these three elements.

The number of cost-effectiveness studies on health-promoting interventions in the older population is also limited. A scoping review [83] performed in the Nordic context identified four cost-effectiveness studies conducted in face-to-face interventions. None of these studies had evaluated remote interventions, but such solutions were suggested as a promising option to support intervention reach and cost-effectiveness [83]. To date, no published studies have evaluated the cost-effectiveness of remote person-centred health promotion to frail older persons. However, a recent systematic review [86] demonstrated an urgent need to assess the cost-effectiveness of remote health services for sub-populations of older adults.

IHOPe is expected to enhance frail older people’s involvement in their care and QoL through person-centred preventive care and health-promoting support through accessible welfare technology.

ICEpop CAPability measure for Older people (ICECAP-O), Activities of Daily Life (ADL), Living with Medicines Questionnaire version 3 (LMQ-3), Tilburg Frailty Indicator (TFI), Clinical Frailty Scale (CFS).

## Data Availability

The datasets generated and/or analysed during the current study are not publicly available due to the study is on the recruitment of participants and data sampling phase but are available from the corresponding author on reasonable request.

## List of abbreviations

IHOPe: Integrating Health Promotion with and for Older People - eHealth
PCC: Person-Centred Care
HCP: HealthCare Professional
RCT: Randomised Controlled Trial
MRC: Medical Research Council
FRESH: Frail Elderly Support Research Group
RN: Registered nurse
VEGA: Name of the register
SPDR: The Swedish Prescribed Drug Register
GSES: The General Self-Efficacy Scale
EQ-5D: Euro-Qual-5 dimension
EQ-VAS: Euro-Qual Visual Analogue Scale
VAS: Visual Analogue scale
HRQoL: Health-Related Quality of Life
ICECAP-O: ICEpop CAPability measure for Older people
QoL: Quality of Life
ADL: The Activities of Daily Life
P-ADL: The Personal Activities of Daily Life
I-ADL: The Instrumental Activities of Daily Life
ICER: Incremental Cost-Effectiveness Ratio
QALY: Quality-Adjusted Life Years
DRG: Diagnosis-Related Group weights
LMQ-3: The Living with Medicines Questionnaire version 3
TFI: The Tilburg Frailty Indicator
CFS: The Clinical Frailty Scale
OR: Odds Ratio
CI: Confidence Interval
CALY: Capability-Adjusted Life Years
